# Supplementary material for: Lactobacilli as a Vector for Delivery of Nanobodies against Norovirus Infection
Source: Pharmaceutics. 2022 Dec 25;15(1):63. doi: 10.3390/pharmaceutics15010063 (PMC9863548; doi:10.3390/pharmaceutics15010063)
Supplement: Supplementary file 1 [file pharmaceutics-15-00063-s001.zip › pharmaceutics-2069149-supplementary.pdf]

Table S1. Bacterial strains and plasmids used in this study.

Strains or plasmids	Characteristics	Source
Strains		
E. coli DH5α	F⁻ φ80*lacZ*ΔM15 Δ(*lacZYA-argF*) *U169 recA1 endA1 hsdR17* (rK⁻, mK⁺) *phoA supE44* λ⁻ *thi-1 gyrA96 relA1*	Invitrogen
L. paracasei BL23	Previously known as *L. zeae* ATCC 393 pLZ15⁻	[13]
L. paracasei BL23-pIAV7	*L. paracasei* BL23 harboring pAF900	[19]
L. paracasei BL23-1E4	*L. paracasei* BL23 harboring pAF900-1E4	This study
Plasmids		
pIAV7	Broad-range vector, Erm, *lacZ*, pWV01 replication origin	[17]
pAF900	Surface-anchored (anchor domain 243-aa PrtP of *L. paracasei* BL23) scFv anti-SAI/II in pIAV7	[19]
pAF900-1E4	pAF900 inserted with 1E4 expression cassette	This study

Table S2. ***Lactobacillus paracasei*** **BL23-pIAV7 (negative control) or BL23-1E4 collected from the feces of germ-free mice after oral administration**

	BL23-pIAV7	BL23-1E4
8 h	5.14×10^6 cfu/ml $\pm 2.08 \times 10^5$	5.53×10^5 cfu/ml $\pm 1.38 \times 10^5$
72 h	9.94×10^6 cfu/ml $\pm 4.38 \times 10^5$	3.14×10^6 cfu/ml $\pm 4.98 \times 10^5$
240 h	3.13×10^5 cfu/ml $\pm 1.31 \times 10^5$	1.87×10^5 cfu/ml $\pm 1.08 \times 10^5$

Samples of feces (0.1 g) collected from germ-free mice at the indicated times after their oral administration *with L. paracasei* BL23-pIAV7 or BL-23-1E4 were suspended in 1 ml PBS and then further diluted 2000-fold in PBS; 100 µl of this final dilution was spread on a MRS agar plate containing 5 µg/ml erythromycin agar plate and grown anaerobically for about 48 h at 37°C. Each data are shown colony forming unit (cfu) per ml of sample. The experiment was performed three times, and results are shown as mean ±1 SD. No *L. paracasei* BL23-pIAV7 and BL-23-1E4 were found at 336 h after oral administration.
